# Evaluation of an OSCE’s implementation and a two-step approach for a theoretical and practical training program in Obstetrics and Gynecology

**DOI:** 10.3389/fmed.2023.1263862

**Published:** 2023-12-21

**Authors:** Ruben Plöger, Alina Abramian, Eva Katharina Egger, Alexander Mustea, Nicole Sänger, Hannah Plöger, Eva Weber, Ulrich Gembruch, Adeline Walter, Brigitte Strizek, Florian Recker

**Affiliations:** ^1^Department of Obstetrics and Gynecology, University Hospital Bonn, Bonn, Germany; ^2^Department of Senology, University Hospital Bonn, Bonn, Germany; ^3^Department of Gynecology and Gynecological Oncology, University Hospital Bonn, Bonn, Germany; ^4^Department of Gynecological Endocrinology and Reproductive Medicine, University Hospital Bonn, Bonn, Germany; ^5^Department of Neonatology and Pediatric Intensive Care, University Hospital Bonn, Bonn, Germany; ^6^Division of Prenatal Medicine, Gynecological Ultrasound and Fetal Surgery, Department of Obstetrics and Gynecology, University of Cologne, Cologne, Germany; ^7^Department of Obstetrics and Perinatal Medicine, University Hospital Bonn, Bonn, Germany

**Keywords:** OSCE, implementation, interprofessional training, undergraduate medical education, national curriculum reform, transition, gynecology and obstetrics

## Abstract

Objective structured clinical examination (OSCE) is a well-known assessment method to evaluate clinical skills and competence in healthcare. Following the recently reformed National Competence-Based Catalog of Learning Objectives in Medicine, the implementation of this assessment method in the training program for medical students is now obligatory in Germany. This major change requires a reorganization not only of the training programs but also of the students themselves and the way they learn. We performed a poll evaluating the students’ opinions regarding these major changes and the implementation of the OSCE with a new training program. To implement this assessment method and to evaluate the OSCE, Kern’s six-step approach comprising (1) problem identification and general needs assessment, (2) needs assessment of the targeted learners, (3) goals and objectives, (4) educational strategies, (5) implementation, and (6) evaluation and feedback was applied. To evaluate and gather feedback, a poll was used to analyze the student’s opinions regarding OSCE in gynecology and obstetrics and OSCE in general, in addition to the regular analysis of the students’ results. To reform the educational strategy, a two-step approach was developed: First, the students completed the regular training program and a written examination, and second, they participated in a 1-week clerkship, in small group teaching, and in the OSCE. The OSCE stations were developed primarily based on the National Competence-Based Catalog and the German Catalog of Learning Objectives in Medicine, as well as on the feedback of experts reflecting their expectations for physicians beginning their careers. The students performed well in the OSCE and gave positive feedback regarding this examination method. Furthermore, they welcomed the upcoming changes by considering OSCE a valuable assessment tool, and they showed appreciation for the two-step approach by supporting the combination of an OSCE and a written examination. Thus, this article presents the implementation of an OSCE and a strategy for the adaptation of the curriculum to fulfill the new OSCE requirements and—to our knowledge—reveals students’ primary opinions regarding the changes in their medical training program for the first time.

## 1 Introduction

The objective structured clinical examination (OSCE) is a method to evaluate the examinee’s clinical skills and competency (1). An OSCE often has numerous stations each with a task, for example, obtaining a patient’s history, which the student conducts by acting as a physician. An examiner evaluates the student by considering certain predefined factors such as the student’s introduction or demeanor. As a result, the OSCE provides greater impartiality and equity than alternative approaches to practical assessments (2). The students are asked to apply knowledge in a situation likely to occur in a physician’s daily work routine instead of simply demonstrating the memorization of facts, as is appraised by most traditional written examinations (3). Therefore, the OSCE allows for a clinical competence-based assessment at a high level of Miller’s pyramid (4). In the last few decades, OSCEs have become a popular assessment method used in almost every medical discipline, such as dentistry (5), and in other healthcare professions such as nursing (6) or midwifery (7). Therefore, the objectives of OSCEs are adapted to each discipline. In obstetrics and gynecology, the reported OSCE includes topics covering a wide spectrum of clinical skills, such as a pelvic examination (8, 9), simulation of delivery (10), examination of a vaginal wet mount to diagnosing vaginitis and/or a sexually transmitted disease (8), and a breast examination (8), and assesses competencies such as the interpretation of cardiotocograph or laboratory results, for example, reflecting ovarian failure (11), management of risk factors and their complications in pregnancy (12), or the treatment and handling of a miscarriage (13). Virtual OSCEs have recently been described as a response to the COVID-19 pandemic (14). As a result, multiple OSCE stations depicting the breadth of the obstetric and gynecologic disciplines have been recorded.

In Germany, the National Competence-Based Catalog of Learning Objectives in Medicine is being reformed in line with the “Master Plan for Medical Studies 2020” (15, 16). In the revised catalog, OSCEs are required as an assessment instrument. As a result, several colleges have begun to implement OSCEs: for example, the Faculty of Medicine at the Goethe University in Frankfurt and the University of Dresden both provide OSCE preparatory courses. Other universities, such as the Friedrich-Alexander University in Erlangen’s Faculty of Medicine and the Charitè in Berlin, have changed their didactical conceptions to a more skill-related model (17).

This study focuses on (1) the development and implementation of an OSCE in gynecology and obstetrics in line with this new reform as a model for other training programs and (2) the reaction of the students regarding the OSCE implementation in their curriculum. Thus, an OSCE in gynecology and obstetrics was developed and implemented using Kern’s six-step approach (18), and a poll was taken to assess the students’ attitude toward the established changes. A two-step approach was developed as an educational strategy to prepare the students for the OSCE (19). The students’ achieved adequate results in the OSCE and their feedback was positive. Regarding their reaction to the overall OSCE implementation in their curriculum, the students welcomed OSCE as a good assessment tool in general and specifically in obstetrics and gynecology. The two-step approach seems to be a suitable educational strategy, supported by the students’ feedback that the combination of a written examination and an OSCE is useful to appropriately assess their knowledge. Overall, the students supported the OSCE’s implementation, adapted quickly to the new teaching methods and assessment design, and appeared to benefit from the OSCE in general.

## 2 Methods

**Figure 1:**
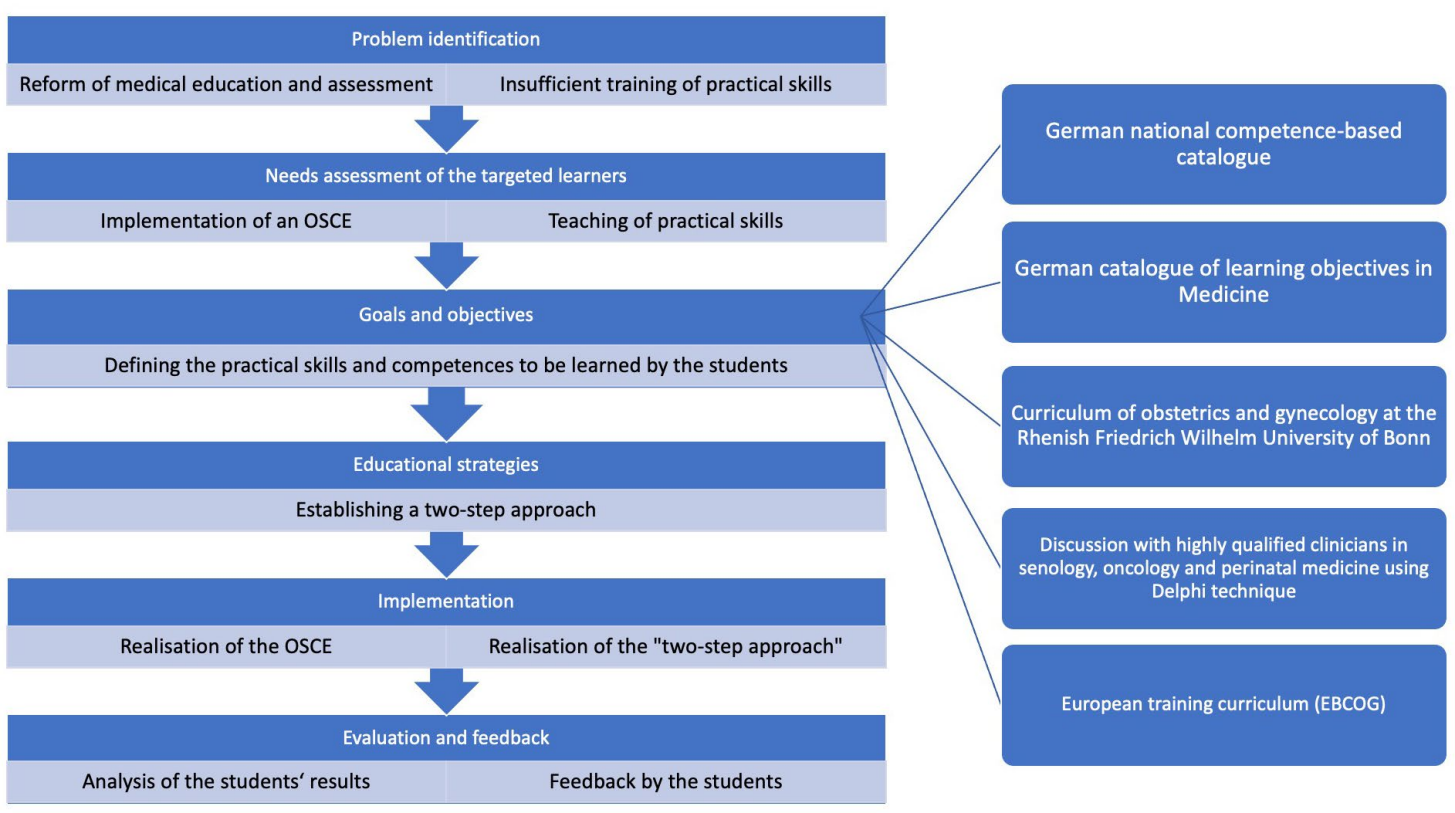
Overview of Kern’s six-step approach applied for OSCE implementation.

To implement an OSCE in the module “Obstetrics and Gynecology” we used Kern’s six-step approach (18). Furthermore, the Delphi technique (20) was used to allow structured communication between highly qualified clinicians in senology, oncology, and perinatal medicine. The six-step approach was applied as follows (Figure 1):

### 2.1 Problem identification

The first step, named “problem identification,” was used to describe the current situation of medical education and to identify the problem. In the past, studying human medicine has been focused on simply learning facts, rather than applying the knowledge in a practical way. Therefore, the recently formed “Master Plan for Medical Studies” now suggests a reform of medical education and practical training in all fields, including obstetrics and gynecology (15). The described changes in the “Master Plan for Medical Studies” were analyzed and compared with the current situation to identify the problem at hand.

### 2.2 Needs assessment of the targeted learners

We assessed the needs of the targeted learners. In this case, the needs of those affected by the upcoming reform in obstetrics and gynecology were discussed and identified.

### 2.3 Goals and objectives

The goals and objectives should be measurable and specify the cognitive, affective, and psychomotor skills necessary to fulfill the defined requirements for the targeted learners and to solve the identified problem. The updated German National Competence-Based Catalog (16) and German Catalog of Learning Objectives in Medicine (21) defined diseases and skills within obstetrics and gynecology to be known and mastered by every student after successful graduation. Thus, the National Competence-Based Catalog and the German Catalog of Learning Objectives in Medicine were analyzed (16, 21). In the next step, the curriculum of obstetrics and gynecology at the Rhenish Friedrich Wilhelm University of Bonn was studied and compared with the requirements of both catalogs to identify discrepancies. Furthermore, the European Board and College of Obstetrics and Gynecology (EBCOG) examinations were evaluated regarding their content. Finally, clinical members with high qualifications in senology, oncology, and perinatal medicine were involved in defining key practical skills and competencies in obstetrics and gynecology using the Delphi technique (20).

### 2.4 Educational strategies

In the next step “educational strategies,” the curriculum was revised to fulfill the educational objectives. Therefore, educational methods were discussed and conceptualized to adapt to the content and structure of the reformed curriculum.

### 2.5 Implementation

In this step, the implementation process of the OSCE after the realization of the curriculum changes was described.

### 2.6 Evaluation and feedback

Finally, the OSCE results and the feedback regarding the implementation of the OSCE were considered and supplemented by questions reflecting the student’s overall opinion of OSCE as an assessment method. The students’ OSCE results provided information regarding the quality of their practical and theoretical preparation for passing the OSCE. The poll with the questions was realized directly after the students had completed the OSCE. The organization committee handed the questionnaires out to the students who voluntarily answered the questions in a separate room. The questionnaires were anonymously collected in a box inside the room. The Ethics Commission of the University Hospital Bonn approved the approach (138/23).

## 3 Results

### 3.1 Problem identification

Prior to the reform, the students’ training program for gynecology and obstetrics at the University Hospital Bonn consisted of lectures and seminars, followed by a written multiple-choice examination, and an internship on the wards, which students had to pass with an oral examination. In this oral examination, the students were asked to present a patient’s history, which is clearly a vital component of a physician’s daily practice. However, emphasis on practical skills and clinical competence was therefore inadequate in this training programm.

The “Master Plan for Medical Studies 2020” led to changes in the German National Competence-Based Catalog (16) and the German Catalog of Learning Objectives in Medicine (21) with more focus on the practical skills and competence that should be mastered by students. Therefore, the reform required an adaption of the current curriculum. Based on this new focus on practical skills and competencies, modified assessment methods, such as the OSCE, were needed, which allow an evaluation of the examinee’s clinical skills and competency (1).

### 3.2 Needs assessment and targeted learners

In this case, the targeted learners were current medical students at the university hospital Bonn, who were enrolled in the module “gynecology and obstetrics” and thus mainly in the 10th semester. Two needs of the targeted learners were assessed: (1) Based on the reform, the students will have to face OSCE as a new assessment method in final examinations (15, 16). Therefore, the students should be confident prior to facing the new method and thus should be trained appropriately, so that the students could focus on the content of the OSCE station, instead of concerning themselves with the new examination setup. (2) As future physicians, the students needed to train and master their practical skills and their competencies. Therefore, students needed modules and clerkships, during which they could train their skills and competencies on models or on patients under supervision. In order to do justice to the students’ needs, the training program had to evolve.

### 3.3 Goals and objectives

Several functions of a physician were mentioned in the German Catalog of Learning Objectives in Medicine (21), with the third description referring to the physician as a communicator (s. A 1.5). This high communication rating had not found a home in educational approaches thus far. Furthermore, the catalog emphasized the importance of medical students being able to gather patients’ histories and execute examination methods (s. A 2.1.1, 2.4.1, 2.5.1, or 2.6.1). The National Competence-Based Catalog of Learning Objectives (16) also requires similar skills (III.8). Thus, successful students should be able to perform these skills during an assessment such as OSCE. In an OSCE, skills would be tested in regard to a certain subject and/or pathology, so that subjects had to be defined. Subjects for the OSCE were picked by overlapping topics from both catalogs (16, 21). For example, the station “speculum insertion” was created based on the listing of the disease: “cervix carcinoma” in the National Competence-Based Catalog of Learning Objectives [VI.06-01.4.12, (16)] and in the German Catalog of Learning Objectives in Medicine [Teil C:53 Krankheitsbilder (21)]. Furthermore, this station covers the item “prevention and screening” from the German Catalog of Learning Objectives in Medicine [Teil A, 2.6 (21)]. In the next step, the curriculum of obstetrics and gynecology at the Rhenish Friedrich Wilhelm University of Bonn and the content of the European Board and College of Obstetrics and Gynecology (EBCOG) examinations were studied and compared with the subjects of the catalogs. Following this, clinical members with high qualifications in senology, gynecological oncology, gynecological endocrinology, reproductive medicine, and perinatal medicine were involved in the process using the Delphi technique (20) to define key practical skills and competencies in obstetrics and gynecology and thus choose the subjects of the OSCE’s stations (s. Table 1). Finally, using the gathered data and professional input, practical skills and competencies in obstetrics and gynecology were chosen, including breast and vaginal examination, delivery supervision, and medical care during pregnancy (s. Table 1). In the station “delivery,” where the Apgar score should be determined, an interdisciplinary approach was established with colleagues from the Department of Neonatology and Pediatric Intensive Care (s. Table 1) in the respect to the subjects of neonatology in the catalogs [VIII.7. (16)].

**Table 1:** Overviews of the stations, their affiliations to each department and the assessed skills and competence.

Stations	Breast cancer	Cervical Neoplasia	Delivery	Gestational diabetes	Preeclampsia
Departments	Senology	Gynecological Oncology	Obstetrics and pediatrics	Obstetrics	Obstetrics
Skills and competencies	Taking patient’s history	Taking patient’s history		Taking patient’s history	Taking patient’s history
	Breast examination	Vaginal examination Performing cervical smear Analysis of the smear	Vaginal examination Guiding a delivery Performing a first care of the newborn	Analysis of the pregnancy record Analysis of a cardiotocogram	Analysis of the pregnancy record Analysis of a cardiotocogram
	Report of the patient’s history and result of the examination	Defining Pap-type	Taking the Apgar score	Diagnose gestational diabetes	Diagnose preeclampsia

### 3.4 Educational strategies

As an educational strategy, a two-step approach was implemented (19): In the first step, the module “obstetrics and gynecology” had to be passed by the students during their third clinical year. In the module, the students could attend lectures and seminars and had to pass a written examination. As the second step, the students had to attend a 1-week clerkship with an OSCE as an assessment tool. During the clerkship, the students rotated within the department, with each student assigned to a different physician each day. Thus, each day, the students were exposed to a specific region such as the prenatal diagnostic department, to a functional area such as the operating room or labor ward, or to patient wards such as the postnatal ward. During the clerkship, consultants from our clinic’s various departments taught the students in small groups (up to 10 students). Vaginal examination, breast examination, birth mechanics, and contraception were among the topics covered. The consultants used models for training as much as possible, so that the students could perform vaginal and breast examinations as well as learn the hand grips important during labor. During the 1-week clerkship, these practical abilities might be honed further with training from the assigned physicians and separately with models in the teaching facility.

Furthermore, the students could use the e-learning platform Amboss^®^ or watch video podcasts reviewing topics such as “breast examination” and “concept of the pregnancy record” to accompany the training program.

### 3.5 Implementation

The OSCE was performed during the last term of medical school. The students (113 students) completed the module “obstetrics and gynecology” and a 1-week clerkship at the Department of Obstetrics and Gynecology prior to the OSCE, as described with two-step approach (s. above). For the OSCE, students were randomly assigned to one of 13 total exam groups. Every group had to travel through three of the five stations, being assigned again at random. Each station lasted 5 min. Students had time between stations to read the case vignette and take a breather before moving on to the next station. Examiners were required to grade students using a digital standardized questionnaire. The examiners received training to familiarize themselves with the digital grading tool prior to the OSCE. Students could reach a maximum of 25 points by fulfilling a checklist of items (22, 23). The tests were carried out on models.

In the station “senology,” students were asked to envision themselves as physicians in a senologic outpatient clinic, where they should take a patient’s medical history, perform an examination, describe a clinical report, and mention one key differential diagnosis. The station “speculum insertion” asked that the examinee obtain the patient’s history, perform a speculum insertion, perform a cervical smear and Papanicolaou’s test, and discuss the clinical results. In the station “delivery,” students were asked to identify the phases of labor of a patient during delivery, to guide the delivery itself using a model, and to give first aid to the infant, which included calculating the Apgar score. In the station “gestational diabetes,” students were confronted with a patient complaining of typical symptoms of this disease. The students were asked to identify their suspected diagnosis, outline the risk factors in the pregnancy record, and describe the cardiotocograph. In the station “preeclampsia,” students had to identify the most likely diagnosis based on the pregnancy record and the patient’s symptoms, indicating a hypertensive disorder during pregnancy. Finally, the students were asked to analyze the patient’s cardiotocograph (s. Table 1).

### 3.6 Feedback

**Figure 2:**
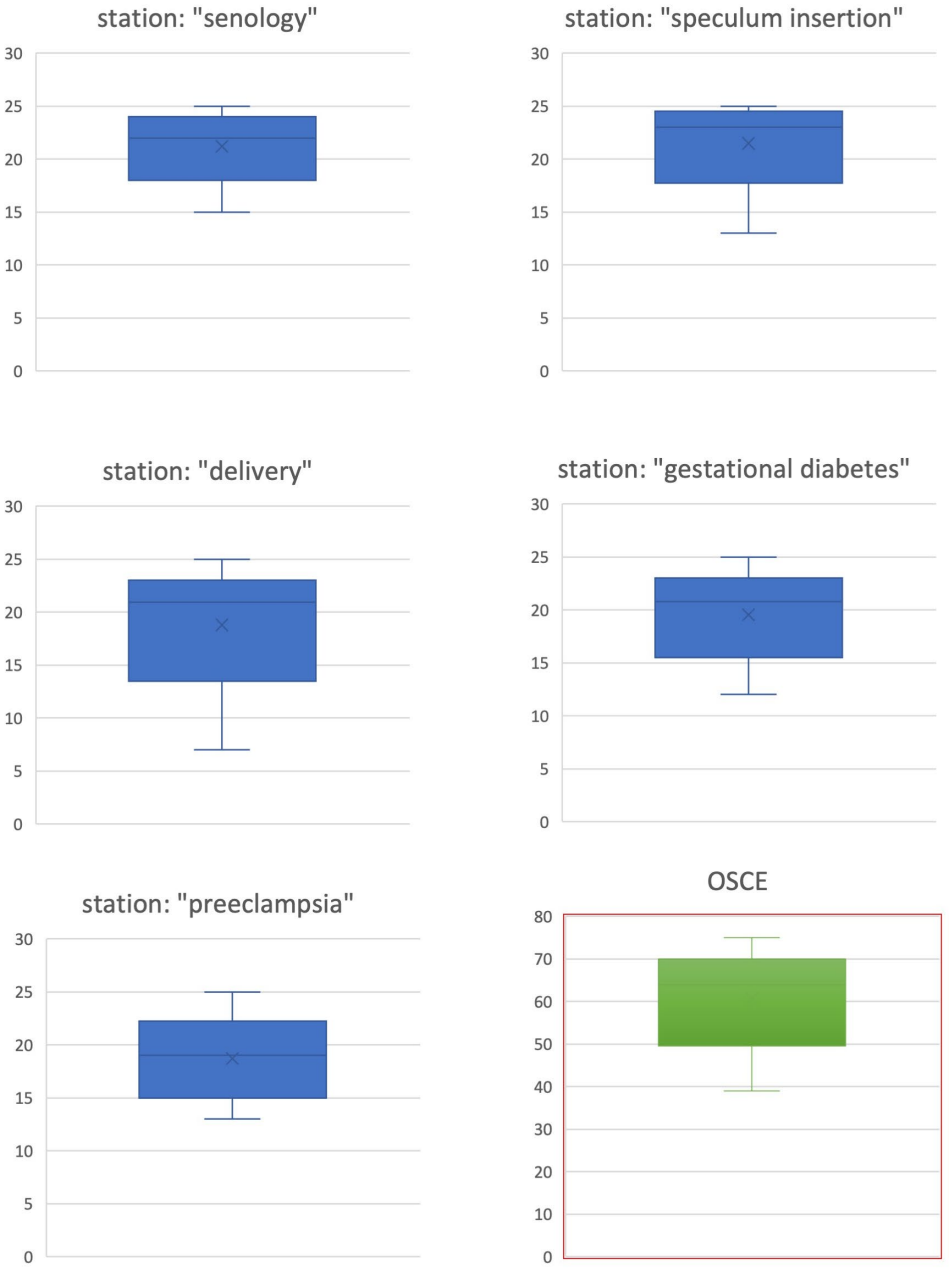
Overview of the OSCE results (box plots).

In total, 113 students had scores ranging from 39 to 75, with a maximum score of 75, resulting in an average score of 63.92. (IQR: 60–69). The average, maximum, and minimum scores for the 58 students who had completed the station “senology” were 21.97 (IQR: 21–23), 25, and 15, respectively. A total of 98 students achieved average, maximum, and minimum scores of 22.41 (IQR: 21–24), 25, and 13, respectively, at the station “speculum insertion.” A total of 80 students completed the “delivery” station and received scores ranging from 7 to 25, with an average of 20.91 (IQR: 20–23). The station “gestational diabetes” was completed by 58 students with an average score of 20.75 (IQR: 19–24), a minimal score of 12, and a maximal score of 25. A total of 45 students passed through the station “preeclampsia” and achieved an average score of 19.47 (IQR: 17–22), a minimal score of 13, and a maximal score of 25 (s. Figure 2).

**Figure 3:**
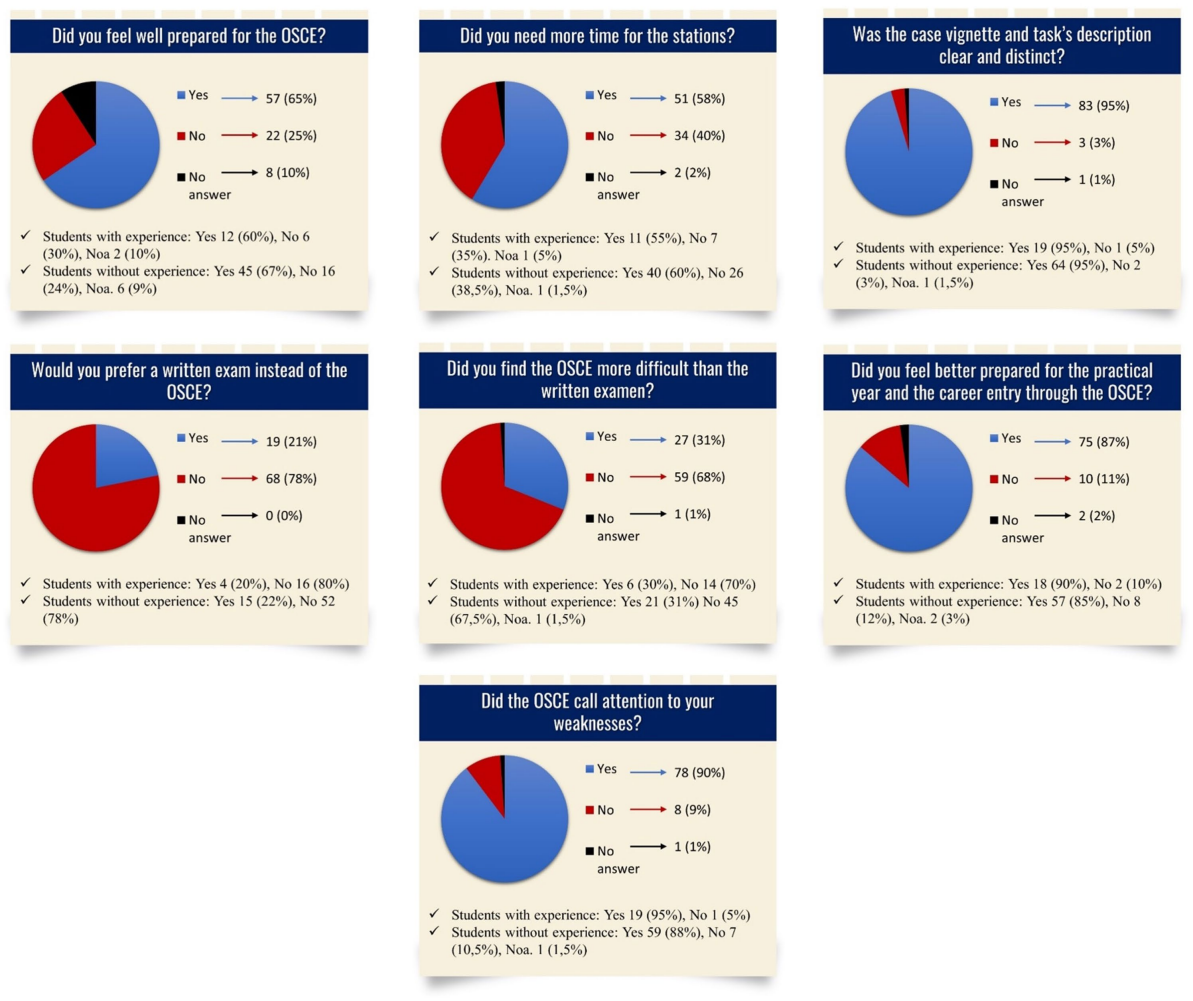
Questionnaire and results regarding the OSCE, Noa: No answer.

**Figure 4:**
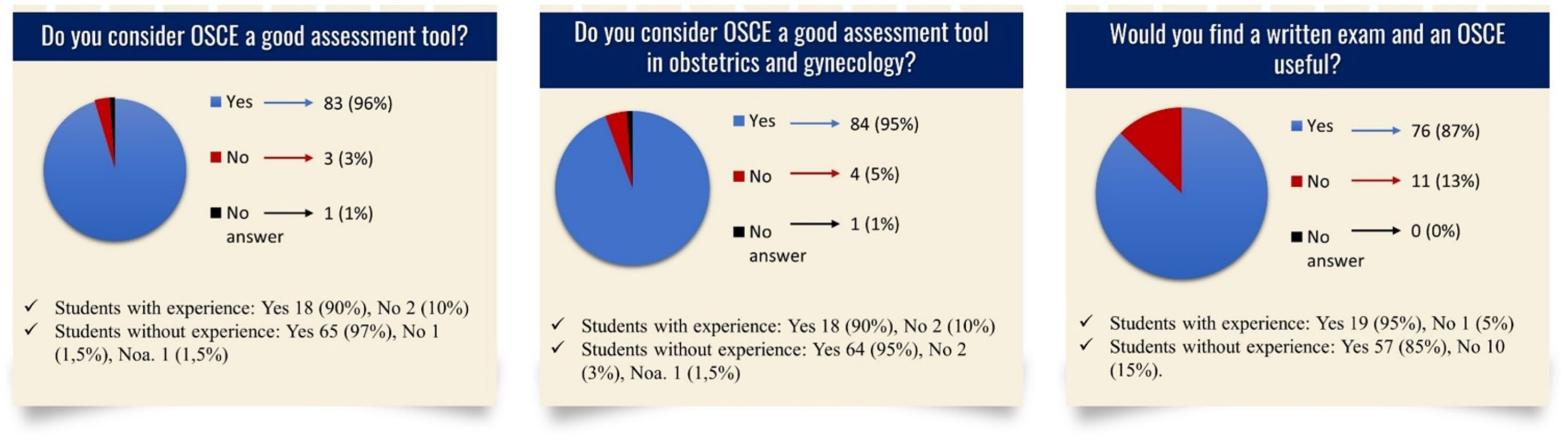
Questionnaire and results regarding OSCE in general, Noa: No answer.

A total of 87 students who completed the OSCE (76.99%) answered the questionnaire (s. Figures 3, 4). The average age was 25 years (IQR: 24–26, youngest student: 23, oldest student: 35). There were 60 female students, 26 male students, and 1 gender-diverse student. In total, 20 students had work experience in healthcare prior to studying medicine, while 67 students had none. There was no statistically significant difference in the replies to these yes/no questions between the groups with and without experience. Figures 3, 4 depict the questions and responses of the students. Open inquiries on the positive and negative elements of the stations revealed the following aspect. Regarding the use of models, the students appreciated the utility of the breast model with the ability to inspect breast lumps in the station “breast examination,” while the students complained that the model in the station “speculum insertion” was insufficient to demonstrate the investigation of the vagina.

## 4 Discussion

The OSCE was implemented in the current curriculum by applying Kern’s six-step approach. Through the students’ experiences of completing the OSCE, they developed a positive attitude toward this specific OSCE and toward OSCE in general, reflected by the poll data.

Kern’s six-step approach is common for curriculum development worldwide (24–26) and has been recently used to implement new topics from the German National Competence-Based Catalog of Learning Objectives in Medicine (16), such as: “conflicts of interest and communicating risk” (27). Further tools to design health professional education curricula are emerging: One approach to modifying the curriculum is the “system thinking perspectives,” by which the educator using the 3P-6Cs toolkit is able to understand the students’ perspectives of the integration of elements within the medical education program and the impact of these elements for the lifelong practice of the student (28). Another approach applies the “twelve tips”: (1) identify the purpose and scope of change, (2) create a vision, (3) develop a strategy for change involving key stakeholders, (4) importance of quick visible wins and communication, (5) analyze the internal environment and culture, (6) consider the external environment, cultural contexts, and political influences, (7) choose the right combination of approaches to change, (8) use project management techniques for operational planning and implementation, (9) acknowledge the psychological impact of changes, (10) plan for transition and loss of competence, (11) do not underestimate the complexity, and (12) celebrate success and shift from project to “New reality” (29). These approaches are based on different priorities such as the establishment of support in a department for the upcoming changes or focus on the student’s perspectives. Kern’s six-step approach was chosen based on its known establishment worldwide, its production of valid and reliable results, and its straightforward structure.

The development and implementation process of an OSCE in the revised curriculum using Kern’s six-step approach was successful based on (1) the satisfactory results of the 113 students passing the OSCE (18, 19), (2) the results of the poll, and (3) the answers to the open inquiries: (1) The comparability with other OSCEs is demonstrated by a similar average result of 85% (10, 11, 19). This may be caused by the similarity of the OSCE stations and the tested skills and competencies required to treat a certain patient profile (8, 9, 11). Only one station included a stark difference to the previously published OSCEs in obstetrics and gynecology: This station devoted to a normal delivery also included the evaluation of the newborn’s health by asking the students to determine an Apgar score. Nonetheless, the different stations seemed to have a similar level of difficulty based on the comparable average results (s. Figure 2).

(2) The response rate of 76.99% in this poll is higher than the overall average response rate of patients and healthcare professionals in surveys worldwide (30), and thus is generalizable based on the standard of many journals (31, 32). The primarily positive answers (65%) to the question: “Did you feel well prepared for the OSCE?” (Figure 3) indicate a well-realized implementation process and support the two-step approach. The negative answers (25%) show the need for further improvement such as the implementation of mock OSCEs (33–35). Students criticized the short amount of time allotted for each station, a comment also found in other OSCE reports (11, 36). From a practical point of view, a short time period for each station allows for the completion of more and various stations in the same time frame, so, in this case, the time length of each station was chosen to last 5 min, similar to most described OSCEs and as recently published for Australian medical schools (37). From the examiner’s point of view, the limited time demands that the student is well prepared and confident in performing the procedure. For an acceptable standard, the students must have practiced the task several times, comparable to the learning curve of surgical procedures [for instance, 80 cases are required for an acceptable standard in laparoscopic colorectal surgery (38)] (39). From the future physician’s view, the limited time reflects the limited time available during a physician’s daily routine. The case vignette and task descriptions of the stations were clear and distinct, which is often described by other studies but may be biased by the one-sided publication of good results (40–43). A total of 78% of the respondents prefer an OSCE, corresponding to studies in Ethiopia and Nigeria (44, 45). A reason for this may be that the students find the OSCE less difficult than written examinations, although this stands in contrast to the fact that published OSCE results show a wide range, some with better and some with worse results in comparison to written assessment (46, 47). On the other hand, 21% of the respondents prefer a written examination, which may be caused by higher stress and nervousness in an OSCE than in a written examination (48). A total of 87% of the respondents feel better prepared for their practical year and their career entry through the OSCE. Dental students described the OSCE as a superior method for evaluating their clinical skills and for presenting questions applicable to real-life clinical situations (43, 49). Furthermore, students attending an OSCE in Oakland show an improvement in long-term knowledge, supporting the benefit of an OSCE for their future careers (10). The students answered that the OSCE as an assessment method called attention to their weaknesses even before receiving the official results. In India, a similar rate of students reported that they could immediately identify their weak spots (50). (3) In the feedback questionnaire, several students reflected the use of models in the OSCE. Because of the ethical difficulties of performing genital examinations on actors, the examinations were realized using models, despite their known limitations. However, the availability of gynecological teaching associates as live models to help teach gynecologic and obstetric examinations in the Netherlands and the United States of America (51–53), seems to be superior to the use of inanimate models (54) as well as provide a better cost–benefit ratio (55).

The establishment of OSCEs as an assessment method in the study of medicine, as required in the National Competence-Based Catalog of Learning Objectives in Medicine, German Catalog of Learning Objectives in Medicine, and the “Master Plan for Medical Studies 2020” (14, 15, 21), seems to be welcomed by the students because questions regarding their perception of OSCE as an assessment tool are mainly answered positively (Figure 4). In others countries, students and teachers have a similarly positive perception of the OSCE (42, 49, 56). This positive perception correlates with known evaluations, in which the students describe OSCE as an accurate measurement method to reflect their knowledge and skills (43, 57, 58). Therefore, the students’ approval rate of OSCE as an assessment tool in obstetrics and gynecology is on a similar level as the approval rate of OSCE as an assessment tool in general (Figure 4) proving the possibility of implementing an OSCE in various medical disciplines (5–7). However, the students attending this OSCE find the combination of written examinations and an OSCE useful. The students may see the need to incorporate both assessment methods to reflect their behavioral and cognitive skills and to be trained in both skills (4, 59), thus supporting the two-step approach. The combination of both assessment methods has the strongest predictive validity of the subsequent performance, correlating with the students’ wishes (60).

One limitation of this study is its design as a single-center study so that the students’ perception of the OSCE is solely based on their learning experience at the University Hospital Bonn. Furthermore, the students’ opinions may be influenced by the OSCE performed shortly before answering the poll. Although 113 students completed the OSCE, only 87 students (76.99%) answered the poll, so possibly, only students with a particular attitude answered the poll. A further limitation of this study is that the poll lacks an analysis of the students’ experiences during the clerkship and the small group teaching. This could be relevant as the students surely had different experiences due to the daily fluctuation of cases during the clerkship and the varying teaching talents of the supervisors, leading to different levels of learning success. This may explain why 25% of students claimed to miss adequate preparation for the OSCE (Figure 3), whereas 75% of students described their preparation as adequate. Furthermore, the conception of the OSCE prevents an analysis regarding internal consistency such as Cronbach’s alpha (61) because only a low number of stations are passed by all students. The realization of more stations for the assessment of the students is hindered by the limited possibility of dispensing physicians from their daily routines. The allocation of three stations from five possible stations allows more aspects of obstetrics and gynecology to be covered despite the shortage of staff.

OSCEs in general and specifically in obstetrics and gynecology are perceived positively by students. The students prefer the OSCE as an additional assessment method, thereby welcoming the changes regarding the implementation of more OSCEs in the study of medicine and favoring the establishment of a two-step learning approach in Germany.

## Data availability statement

The raw data supporting the conclusions of this article will be made available by the authors, without undue reservation.

## Ethics statement

The studies involving humans were approved by Ethics Commission of University Hospital Bonn. The studies were conducted in accordance with the local legislation and institutional requirements. The ethics committee/institutional review board waived the requirement of written informed consent for participation from the participants or the participants’ legal guardians/next of kin because the study is based on a voluntary evaluation of teaching methods without biomedical experiments on humans.

## Author contributions

RP: Conceptualization, Data curation, Formal analysis, Investigation, Methodology, Visualization, Writing – original draft, Writing – review & editing. AA: Conceptualization, Writing – review & editing. EE: Conceptualization, Data curation, Writing – review & editing. AM: Resources, Writing – review & editing. NS: Resources, Writing – review & editing, Data curation. HP: Writing – review & editing, Validation, Writing – original draft. EW: Resources, Writing – review & editing. UG: Resources, Writing – review & editing. AW: Data curation, Writing – review & editing. BS: Data curation, Resources, Writing – review & editing. FR: Conceptualization, Data curation, Formal analysis, Funding acquisition, Investigation, Methodology, Project administration, Resources, Supervision, Writing – original draft, Writing – review & editing.
